# Determinants of Modern Contraceptive Use among Married Women of Reproductive Age in a Municipality of Nepal: A Cross-Sectional Study

**DOI:** 10.3126/nje.v16i2.91901

**Published:** 2026-08-03

**Authors:** Samikshya Acharya, Satya Raj Upadhayay

**Affiliations:** 1 School of Public Health and Community Medicine, B.P. Koirala Institute of Health Sciences, Dharan, Nepal; 2 Central Department of Rural Development, Faculty of Humanities and Social Sciences, Tribhuvan University, Kirtipur, Kathmandu, Nepal

**Keywords:** Family planning, Modern contraceptives, Married women, Nepal, Reproductive health

## Abstract

**Background:**

The use of modern contraceptives plays a crucial role in reducing unintended pregnancies and improving maternal and child health outcomes. The level of contraceptive use is strongly linked to the total fertility rate (TFR) and population growth rate. This study aimed to assess the utilization of modern contraceptive methods and associated factors among married women of reproductive age (MWRA) in Birendranagar Municipality, Surkhet district.

**Material and Methods:**

A community-based cross-sectional study was conducted from April 28, 2023 to October 31, 2023. Eight wards were selected by simple random sampling from the 16 wards of Birendranagar Municipality. The total MWRA population (25,738) in the selected wards was identified, and sample allocation was done using probability proportional to size (PPS). Within each ward, households were selected by systematic random sampling. A sample size of 494 was calculated using the Cochrane single proportion formula. The validated NDHS 2022 questionnaire was used for data collection through face-to-face interviews. Cronbach's alpha was used to assess reliability. Data were analyzed using SPSS version 11.5. Descriptive statistics, Chi-square test, and binary logistic regression were performed at a 95% confidence interval (CI).

**Result:**

The mean (± SD) age of participants was 32.97 ± 7.791 years. All respondents (100%) had previously used a family planning method, and 70.6% were currently using modern contraceptives. The most frequently used method was the male condom (29.2%). Total unmet need for modern contraceptives was 22.8%. Multivariate analysis showed that Janjati ethnicity (AOR=4.0, 95% CI: 1.3–12.1), husband's occupation as farmer/daily wage worker (AOR=8.4, 95% CI: 3.7–19.1) or engaged with work (AOR=8.3, 95% CI: 4.5–15.2), and information about side effects provided at the healthcare facility (AOR=1.9, 95% CI: 1.2–3.3) were significantly associated with modern contraceptive use.

**Conclusion:**

Modern contraceptive use was 70.6% among married women of reproductive age in Birendranagar Municipality, higher than the national estimate (43%), while unmet need remained at 22.8% which is almost similar to the national data (21%) [1]. These findings highlight the need to improve counselling quality and implement targeted interventions to reduce unmet need and promote informed contraceptive use.

## Introduction

Making family planning services universally accessible has been a cornerstone of the global development agenda. The International Conference on Population and Development Program of Action declared family planning a fundamental human right. Target 3.7 of the Sustainable Development Goals (SDGs) emphasizes universal access to sexual and reproductive health-care services, including family planning. Despite this, access to and use of modern contraceptives remains low in low- and middle-income countries like Nepal [2].

The utilization of modern contraceptives is shaped by several intersecting factors including socioeconomic status, educational attainment, geographic location, cultural norms, and individual psychology. Age of the woman, number of existing children, desired family size, and attitude toward family planning are all known to influence contraceptive uptake. Improving utilization of modern contraceptive methods is critical to reducing maternal and child mortality and to promoting the socio-economic development of communities and the nation as a whole [3].

The utilization of contraceptive devices can prevent unwanted pregnancy, early childbearing, and abortion related death. Although the knowledge of family planning is almost universal among adolescent and youth, the use of modern contraceptive is still very low among young women. Antenatal Care (ANC) is one of the key components to monitor and reduce the risk of mortality among young mothers during pregnancy and childbirth. Similarly, skilled birth attendance (SBA) is another important factor in reducing maternal and neonatal mortality [4]. According to the World Family Planning 2022 report, of the 1.9 billion women of reproductive age, approximately 874 million use modern contraceptives, while 92 million rely on traditional methods. However, 164 million women who wish to delay or prevent pregnancy do not use contraception and are thus categorized as having unmet need. The proportion using modern methods increased from 35% in 1990 to 45% in 2021 [5].

According to NDHS 2022, the contraceptive prevalence rate (CPR) among currently married women of reproductive age in Nepal is 53%, with 43% using any modern method. The unmet need for family planning has declined from 32% in 1996 to 21% in 2022. Karnali Province, where this study was conducted, has historically reported lower access to reproductive health services, making the assessment of contraceptive use in this region particularly relevant [6].

Despite widespread awareness of family planning methods in Nepal, actual utilization of modern contraceptives remains suboptimal, particularly in underserved regions like Karnali Province. Understanding the socio-demographic and health service factors influencing contraceptive uptake is essential to address unmet needs and reduce maternal and child health risks. This study aims to assess the prevalence and determinants of modern contraceptive use among married women of reproductive age in Birendranagar Municipality, Surkhet District, Karnali Province, Nepal.

## Methodology

### Study Design and Participants

A community-based cross-sectional study was conducted among married women of reproductive age (15–49 years) residing in Birendranagar Municipality, Surkhet District, Karnali Province, Nepal. The study was carried out from April 28, 2023 to October 31, 2023.

### Study Setting

Birendranagar Municipality is located in Surkhet District, Karnali Province. It serves as both the district headquarters of Surkhet and the capital of Karnali Province. The municipality covers an area of 245.06 square kilometres and comprises 16 wards. According to the 2021 census, its total population is 154,886 [7].

### Inclusion Criteria

Married women aged 15–49 years who were permanent residents of Birendranagar Municipality and who gave informed written consent were included in the study.

### Exclusion Criteria

Women who were temporarily residing in the municipality, those who were unable to communicate, and those who declined to participate were excluded.

### Sample Size Calculation

The sample size was calculated using the Cochrane single proportion formula: n = Z^2^pq/d^2^. Based on a prevalence of modern contraceptive use of 46.1% from a prior study in rural Nepal [8], and assuming 95% CI, 5% margin of error, and 10% non-response rate, the final sample size was 494.

### Sampling Technique

Eight wards were selected from the 16 wards of Birendranagar Municipality using simple random sampling. The total MWRA population (25,738) in the selected wards was identified. Sample was then allocated to each ward using probability proportional to size (PPS). Within each ward, households were selected using systematic random sampling. The sampling interval was calculated by dividing the total number of households in each ward by the allocated sample size, with the starting household selected randomly. Table 1 shows the ward-wise distribution of the sample.

### Data Collection

Data were collected using a validated NDHS 2022 questionnaire through face-to-face interviews. Participants' queries regarding family planning were answered after the completion of the data collection process on the same day. Individuals reporting side effects from contraceptives were encouraged to seek assistance at the nearest healthcare facility.

### Questionnaire Design and Validation

The NDHS 2022 questionnaire was used to maintain the validity of the study. The questionnaire was pre-tested among 10% of the similar sample size in an unselected ward of Birendranagar Municipality. After pre-testing, necessary modifications were made based on expert feedback. Cronbach's alpha was computed to assess internal consistency and reliability of the questionnaire.

### Outcome Variable

The primary outcome variable was current use of modern contraceptive methods among married women of reproductive age (yes/no).

### Explanatory Variables

Explanatory variables included age, education level, husband's education, ethnicity, religion, occupational status of respondent and husband, family type, annual household income, age at marriage, fertility history, knowledge of family planning, access to counseling at health facilities, FCHV visits, and ANC/PNC/EPI service utilization.

### Ethical Committee Approval

Ethical clearance was obtained from the Institutional Review Committee of B.P. Koirala Institute of Health Sciences, Dharan, with reference number 684/079/080-IRC. Permission was obtained from Birendranagar Municipal Office. Informed written consent was taken from all participants before the interview, and confidentiality was maintained throughout the study.

### Data Management and Statistical Analysis

Data were entered into Microsoft Excel, coded numerically, and exported to SPSS version 11.5 for analysis. Data completeness was checked daily, and at least 10% of entries were cross-verified weekly. Descriptive statistics were presented as frequencies, percentages, mean, and standard deviation. Chi-square test was used for bivariate association at 95% CI with significance set at p<0.05. Variables with p<0.2 in bivariate analysis were entered into binary logistic regression for multivariate analysis. Adjusted odds ratios (AORs) with 95% CI were reported to identify independent predictors of modern contraceptive use.

## Results

### Participant Demographics

A total of 494 married women of reproductive age participated in the study. The mean (± SD) age was 32.97 ± 7.791 years. Tables 2, 3, and 4 summarize the socio-demographic characteristics, fertility patterns, knowledge, utilization, and unmet need data of the respondents.

The majority of respondents (42.9%) were aged 25–34 years. Most (96.2%) were literate, with 38.5% having attained secondary level education. Brahmin/Chhetri constituted the largest ethnic group (54.9%), and 85.8% were Hindu. In terms of fertility, most respondents (42.9%) had married between 20–24 years of age. About 70.2% had been pregnant in their lifetime, with most first pregnancies occurring at age 20–24 years (45.5%). The majority (74.6%) had 1–2 children.

All respondents (100%) had heard of at least one family planning method. The most common source of information was health service providers (88.6%), followed by radio (51.9%) and television (44.8%). Awareness of male condoms was highest (91.5%), followed by Depo-Provera (88.2%) and oral contraceptive pills (87.2%). Health posts were identified as the primary facility for family planning services by 83.8% of respondents.

Currently, 70.6% (n=349) of respondents were using modern contraceptives. The most commonly used method was the male condom (29.2%), followed by oral pills (22.1%) and Depo-Provera (13.8%). Among non-users (n=145), the primary reason for non-use was the husband being away for work (51.0%). About 66.6% of facilities had informed respondents about side effects of contraceptives, and 60.3% had provided counseling on what to do if side effects occurred.

Among the non-users of modern contraceptives, 13.1% were currently pregnant and only 5.3% of those pregnancies were unplanned. Of all respondents, 28.8% had not desired their most recent pregnancy. The total unmet need for modern contraceptives was 22.8%, comprising 11.3% unmet need for spacing and 11.5% for limiting. Nearly half of the respondents (49.4%) had no plans for future childbearing.

In the bivariate analysis, ethnicity, husband's occupation, number of male children, information about side effects at facilities, and fieldworker discussion about family planning showed significant associations with modern contraceptive use. Janjati women were nearly five times more likely to use modern contraceptives compared to Thakuri women (COR=4.790, 95% CI: 1.772–12.949). Husbands in farming/daily wage work were 8.6 times more likely to have wives using modern contraceptives compared to migrant workers (COR=8.647, 95% CI: 3.893–19.206). Regarding unmet need variables, respondents with undecided future plans were nearly 22 times more likely to use modern contraceptives compared to those who could not conceive (COR=21.949, 95% CI: 4.847–99.387).

In the multivariate analysis, three variables remained statistically significant: Janjati ethnicity (AOR=4.037, 95% CI: 1.343–12.139, p=0.013), husband's occupation as farmer/daily wage worker (AOR=8.389, 95% CI: 3.685–19.101, p<0.001), husband engaged with work (AOR=8.301, 95% CI: 4.534–15.231, p<0.001), and not being informed about side effects at the facility (AOR=1.987, 95% CI: 1.211–3.262, p=0.007).

## Discussion

### Utilization of Modern Contraceptives

The present study found that 70.6% of married women of reproductive age currently use modern contraceptives. This is higher than the national average of 43% [1] and exceeds findings from comparable Nepali settings, 63.84% in 2021 [9], 56.9% in Rolpa in 2020 [10], and 46.1% in rural Nepal in 2019 [11]. However, it contrasts with a study from specific western Nepal municipalities in 2022, which reported a prevalence of only 11.9%. The relatively higher prevalence in Birendranagar Municipality may reflect its status as a provincial capital, with better health infrastructure and greater awareness of family planning services.

### Knowledge and Source of Information

All respondents (100%) were aware of at least one family planning methodHealth posts were identified as the primary source of family planning information (83.8%), followed by PHC and government hospitals. A survey in Dhading, Nepal in 2020 similarly reported that 85.7% of respondents obtained family planning information from health posts [12]. The proximity of health posts to local communities ensures accessibility and explains their dominant role as information sources.

### Number of Women Having Male Children

The bivariate analysis showed a significant association between the number of male children and modern contraceptive use (COR=2.579, 95% CI: 1.005–6.619, p=0.049). A similar study in Nepal in 2020 also found a significant association between the number of living male children and contraceptive utilization (p<0.001) [4]. This may reflect patriarchal norms where having a male child is perceived as fulfilling reproductive goals, prompting couples to limit further pregnancies through contraceptive use.

### Ethnicity

In the bivariate analysis, ethnicity was significantly associated with modern contraceptive use (p<0.001). In the multivariate analysis, Janjati women had four times higher odds of using modern contraceptives compared to Thakuri women (AOR=4.037, 95% CI: 1.343–12.139, p=0.013). This is consistent with a 2021 Nepal study where Janajati women were approximately two times more likely to use modern contraception than Brahmin/Chhetri women (AOR=2.08, 95% CI: 1.16–3.71) [13]. Differences in healthcare access, awareness, education, and cultural norms among ethnic communities may explain this variation.

### Husband's Occupation

Husband's occupation was a strong and significant predictor of modern contraceptive use in both bivariate and multivariate analyses. Husbands engaged in farming or daily wage work were 8 times more likely to have wives using modern contraceptives than husbands involved in migrant work (AOR=8.389, 95% CI: 3.685–19.101). A similar finding was reported from Kailali district, Nepal in 2020 (AOR=3.2, 95% CI: 2.0–6.0) [14]) and from Bharatpur, Nepal in 2022 (p=0.004) [15]. Physical absence due to migration likely reduces couple communication about family planning and limits joint decision-making on contraceptive use.

### Reason for Non-Use

The primary reason for not using modern contraceptives was the husband's foreign employment (51%), followed by other reasons (31%) such as infertility and menstrual stoppage. This pattern mirrors findings from Kailali district in 2020, where 40.5% cited foreign employment as the primary reason [14]. The physical separation caused by migration creates a practical barrier to contraceptive access and couples' joint engagement in family planning.

### Unmet Need

The total unmet need for modern contraceptives was 22.8%, 11.3% for spacing and 11.5% for limiting. This is comparable to the national unmet need of 21% [1], suggesting the study area is broadly representative of national patterns. Other Nepali studies have reported much higher unmet need: 50% in Kailali in 2020[15] and 54.2% one year after delivery [16]. These differences likely reflect variation in geographic access, education, and socio-cultural context.

### Future Plans for Children

Respondents with undecided or unknown future plans were nearly 22 times more likely to use modern contraceptives compared to those who could not conceive (COR=21.949, 95% CI: 4.847–99.387). A comparable Nepal study in 2020 showed that women wanting more children were less likely to use modern contraceptives (OR=0.57, p<0.001) [4]. The desire to limit family size clearly motivates contraceptive uptake, consistent with the purpose of contraception.

### Limitations

Given the cross-sectional design, it was not possible to establish causal relationships between the utilization of modern contraceptives and the explanatory variables. Perspectives of husbands and healthcare providers were not captured. Recall bias may have occurred during data collection, particularly for variables such as age of first pregnancy, counseling services received during past pregnancies or childbirths, media use, and wealth quintiles.

## Conclusion

Modern contraceptive use among married women of reproductive age in Birendranagar Municipality was 70.6%, considerably higher than the national average of 43%, yet unmet need remained substantial at 22.8%. Utilization was significantly associated with ethnicity (Janjati), husband's occupation, and information about side effects provided at healthcare facilities. These findings point to the need for strengthening the quality of counseling — especially on side effects — and designing targeted interventions for women whose husbands are engaged in migrant work. Equitable access to family planning services and promotion of informed contraceptive choice remain essential strategies to enhance reproductive health outcomes in this region.

### What is already known on this topic

It is well established that modern contraceptive use in Nepal remains below the global average, with significant regional disparities. Husband's education, women's empowerment, economic status, and access to health services are recognized determinants of contraceptive use. Karnali Province has historically lagged behind other provinces in reproductive health indicators.

### What this study adds

This study provides updated, ward-level data on modern contraceptive use from Birendranagar Municipality, Karnali Province. It identifies husband's occupation, specifically migrant work, as a significant barrier to contraceptive use, and shows that information about side effects at healthcare facilities is an independent predictor. These locally relevant findings can inform targeted health programming in the municipality and similar settings across Karnali Province.

### Future scope of the study

Future research should consider longitudinal designs to establish causal relationships. Studies incorporating husbands' and healthcare providers' perspectives would offer a more complete picture. Qualitative studies exploring cultural and community factors influencing contraceptive decision-making would complement these quantitative findings, especially in the context of out-migration.

## Figures and Tables

**Table 1:** Ward-wise distribution of MWRAs

Wards	Ward Population	PPS (Ward Popn/Total Popn × Sample Size)
**3**	5,134	99
**5**	1,826	35
**6**	1,947	37
**7**	2,568	48
**8**	2,100	40
**9**	3,186	61
**10**	5,176	99
**12**	3,901	75
**Total**	**25,738**	**494**

**Table 2:** Socio-demographic Profile and Fertility Pattern of Respondents

Characteristics	Categories	Frequency (n)	Percentage (%)
**A. Socio-demographic Profile**			
**Age (in years)**	**15–24**	136	27.5
	**25–34**	212	42.9
	**35–44**	110	22.3
	**>44**	36	7.3
**Mean ± SD: 32.97 ± 7.791 years**			
**Educational status**	**Literate**	475	96.2
	**Illiterate**	19	3.8
**Literate respondents (n=475)**	**General read and write**	26	5.5
	**Primary**	147	31
	**Secondary**	183	38.5
	**Higher Secondary**	119	25
**Education of husband**	**Literate**	490	99.2
	**Illiterate**	4	0.8
**Literate husbands (n=490)**	**General read and write**	15	3
	**Primary**	110	22.4
	**Secondary**	176	36
	**Higher Secondary**	189	38.6
**Ethnicity**	**Brahmin/Chhetri**	271	54.9
	**Janjati**	139	28.1
	**Dalit**	65	13.2
	**Thakuri**	19	3.8
**Religion**	**Hindu**	424	85.8
	**Buddhist**	3	0.6
	**Muslim**	1	0.2
	**Christian**	66	13.4
**B. Fertility Pattern**			
**Age of marriage (n=494)**	**<15**	16	3.2
	**15–19**	201	40.7
	**20–24**	212	42.9
	**25–34**	65	13.2
**Mean ± SD: 19.06 ± 2.910 years**			
**Age of first pregnancy (n=347)**	**<15**	2	0.6
	**15–19**	137	39.5
	**20–24**	158	45.5
	**25–29**	45	13
	**30–39**	5	1.4
**Mean ± SD: 20.69 ± 3.394 years**			
**Number of children (n=334)**	**1–2**	249	74.6
	**3–4**	76	22.8
	**≥5–6**	9	2.6
**Number of male children (n=278)**	**1–2**	259	93.2
	**3–4**	19	6.8
**Number of female children (n=195)**	**1–2**	183	93.8
	**3–4**	10	5.1
	**5–6**	2	1
**Age of first child (n=334)**	**<1**	24	7.2
	**1–9**	124	37.1
	**10–19**	103	30.8
	**20–29**	72	21.6
	**30–39**	11	3.3
**Mean ± SD: 12.51 ± 8.888 years**			
**Birth interval (n=213)**	**≤1–2 years**	64	30
	**3–4 years**	93	43.7
	**5–6 years**	30	14.1
	**>6 years**	26	12.2

**Table 3:** Knowledge of Family Planning and Utilization of Modern Contraceptive Methods

Characteristics	Categories	Frequency (n)	Percentage (%)
**A. Knowledge of Respondents about Family Planning Methods (n=494)**			
**Heard of family planning**	Yes	494	100.0
**Source of information**	Health service providers	437	88.6
	Radio	256	51.9
	Television	221	44.8
	Friends	121	24.5
	Family	118	23.9
	Newspaper	99	20.1
	Others (Schools, colleges)	2	0.4
**Known family planning methods**	Male condoms	451	91.5
	Depo-Provera	435	88.2
	Oral contraceptive pills	430	87.2
	Implant	362	73.4
	IUCD	310	62.9
	Mini lap	249	50.5
	Vasectomy	235	47.7
	Female condom	186	37.7
	Lactational amenorrhea	48	9.7
	Others (ECPs)	5	1.0
**Facility for FP services**	Health post	414	83.8
	PHC	204	41.3
	Government hospital	192	38.9
	Medical shop	101	20.4
	PHC/ORC	51	10.3
	FCHV	51	10.3
	Marie Stopes	36	7.3
	Private Hospital/Clinic	33	6.7
	FPAN	24	4.9
	Nepal Red Cross Society	15	3.0
**Know how soon pregnancy can occur post-birth**	<1 year	249	50.4
	1–3 years	235	47.6
	4–6 years	6	1.2
	Don't know	4	0.8
**Appropriate spacing between births**	1–2 years	15	3.0
	3–4 years	45	9.1
	5–6 years	392	79.4
	>5–6 years	42	8.5
**B. Utilization of Modern Contraceptive Methods and Related Factors**			
**Ever used family planning (n=494)**	Yes	494	100.0
**Currently using modern contraceptives (n=494)**	Yes	349	70.6
	No	145	29.4
**Reason for non-use (n=145)**	Husband away for long period	74	51.0
	Others (infertility, menstrual stoppage)	45	31.0
	Wants to become pregnant	16	11.0
	Sexual activity not resumed	7	4.8
	Thinks no need to use	2	1.4
	Husband's disapproval	1	0.7
**Method currently used (n=349)**	Male condom	102	29.2
	Pills	77	22.1
	Depo-Provera	48	13.8
	Others	33	9.5
	Vasectomy	26	7.4
	Minilap	26	7.4
	Implant	25	7.2
	IUCD	9	2.6
	Female condom	3	0.9
**Facility for FP services (n=494)**	Health post	190	48.3
	Medical shop	97	24.7
	Government hospital	94	23.9
	FCHV	7	1.8
	FPAN	5	1.3
	Private hospital/clinic	3	0.8
	PHC	3	0.8
	Marie Stopes	2	0.5
**Side effects informed at facility (n=494)**	Yes	329	66.6
	No	165	33.4
**Counseling on side effects (n=494)**	Yes	298	60.3
	No	196	39.7
**Other FP methods told (n=494)**	Yes	257	52.0
	No	237	48.0
**FCHV visit last 12 months (n=494)**	Yes	244	49.4
	No	250	50.6
**Fieldworker discussed FP (n=494)**	Yes	185	37.4
	No	309	62.6
**ANC visit in last pregnancy (n=334)**	Yes	304	91.0
	No	30	9.0
**FP counseling during ANC (n=304)**	Yes	216	71.1
	No	88	28.9
**PNC visit (n=331)**	Yes	281	84.9
	No	50	15.1
**FP counseling during PNC (n=281)**	Yes	212	75.4
	No	69	24.6
**EPI visit (n=333)**	Yes	332	99.7
	No	1	0.3
**FP counseling during EPI (n=332)**	Yes	219	66.0
	No	113	34.0

**Table 4:** Unmet Need of Respondents

Characteristics	Categories	Frequency (n)	Percentage (%)
**A. Unmet Need of Respondents**			
**Currently pregnant (n=145)**	**Yes**	19	13.1
	**No**	126	86.9
**Wanted/unwanted current pregnancy (n=19)**	**Yes**	18	94.7
	**No**	1	5.3
**Pregnancy in lifetime (n=494)**	**Have been pregnant**	347	70.2
	**Have not been pregnant**	147	29.8
**Wanted/unwanted last pregnancy (n=347)**	**Yes**	247	71.2
	**No**	100	28.8
**If unwanted: Later or No more (n=100)**	**Later**	45	45.0
	**No more**	55	55.0
**Waiting period for next child (n=45)**	**1–2 years**	26	57.8
	**3–4 years**	9	20.0
	**5–6 years**	9	20.0
	**>5–6 years**	1	2.2
**Future plan of child (n=494)**	**Want child**	144	29.1
	**No more/No one**	244	49.4
	**Can't conceive**	16	3.2
	**Undecided/Don't know**	90	18.2
**If want child, wait how long (n=144)**	**1–2 years**	40	27.8
	**3–4 years**	44	30.6
	**≥5–6 years**	34	23.6
	**Soon/now**	26	18.0

**Table 5:** Bivariate and Multivariate Analysis of Factors Associated with Modern Contraceptive Use

Characteristics	Categories	Yes n(%)	No n(%)	p-value	COR/AOR (95% CI)	Remarks
**A. Bivariate Analysis: Socio-demographic Variables**						
**Age (years)**	**15–24**	92 (67.6)	44 (32.4)	—	Ref.	**NS**
	**25–34**	149 (70.3)	63 (29.7)	0.603	1.131 (0.711–1.800)	
	**35–44**	80 (72.7)	30 (27.3)	0.388	1.275 (0.734–2.216)	
	**>44**	28 (77.8)	8 (22.2)	0.243	1.674 (0.706–3.971)	
**Educational status**	**Illiterate**	13 (68.4)	6 (31.6)	—	Ref.	**NS**
	**Literate**	336 (70.7)	139 (29.3)	0.117	1.116 (0.416–2.994)	
**Ethnicity**	**Thakuri**	8 (42.1)	11 (57.9)	—	Ref.	**S**
	**Dalit**	36 (55.4)	29 (44.6)	0.311	1.707 (0.607–4.799)	**NS**
	**Janjati**	108 (77.7)	31 (22.3)	0.002	4.790 (1.772–12.949)	**S**
	**Brahmin/Chhetri**	197 (56.4)	74 (51.0)	0.007	3.660 (1.417–9.457)	**S**
**Religion**	**Hindu**	297 (70.0)	127 (30.0)	0.471	Ref.	**NS**
	**Non-Hindu**	52 (74.3)	18 (25.7)	—	1.235 (0.695–2.195)	
**Occupational status**	**Not employed**	80 (31.9)	171 (68.1)	0.212	Ref.	**NS**
	**Employed**	178 (73.3)	65 (26.7)	—	1.281 (0.868–1.890)	
**Husband's occupation**	**Migrant workers**	19 (27.5)	50 (72.5)	—	Ref.	**S**
	**Farmer/Daily wage**	46 (76.7)	14 (23.3)	<0.001	8.647 (3.893–19.206)	**S**
	**Engaged with work**	284 (77.8)	81 (22.2)	<0.001	9.227 (5.150–16.530)	**S**
**Family type**	**Joint**	41 (69.5)	18 (30.5)	0.835	Ref.	**NS**
	**Nuclear**	308 (70.8)	127 (29.2)	—	1.065 (0.589–1.924)	
**Family annual income**	**<2,50,000**	35 (62.5)	21 (37.5)	—	Ref.	**NS**
	**2,50,000–6,00,000**	257 (71.2)	104 (28.8)	0.188	1.483 (0.824–2.667)	
	**>6,00,000**	57 (74.0)	20 (26.0)	0.157	1.710 (0.813–3.595)	
**Age of marriage**	**≤19**	152 (70.0)	65 (30.0)	0.795	Ref.	**NS**
	**>19**	197 (71.1)	80 (28.9)	—	1.053 (0.713–1.555)	
**Having children**	**≤2**	173 (69.5)	76 (30.5)	0.219	Ref.	**NS**
	**>2**	65 (76.5)	20 (23.5)	—	1.428 (0.808–2.522)	
**Having male children**	**>2**	10 (52.6)	9 (47.4)	—	Ref.	**S**
	**≤2**	192 (74.1)	67 (25.9)	0.049	2.579 (1.005–6.619)	**S**
**Having female children**	**≤2**	133 (72.7)	50 (27.3)	0.581	Ref.	**NS**
	**>2**	9 (75.0)	3 (25.0)	—	1.128 (0.293–4.335)	
**B. Bivariate Analysis: Associated Factors**						
**Side effects informed at facility**	**Yes**	215 (65.3)	114 (34.7)	<0.001	Ref.	**S**
	**No**	134 (81.2)	31 (18.8)	—	2.292 (1.459–3.601)	**S**
**FCHV visit in last 12 months**	**No**	167 (66.8)	83 (33.2)	0.058	Ref.	**NS**
	**Yes**	182 (74.6)	62 (25.4)	—	1.459 (0.987–2.156)	
**Fieldworker discussed FP**	**No**	208 (67.3)	101 (32.7)	0.036	Ref.	**S**
	**Yes**	141 (76.2)	44 (23.8)	—	1.556 (1.029–2.353)	**S**
**ANC counseling**	**Yes**	153 (70.8)	63 (29.2)	0.793	Ref.	**NS**
	**No**	61 (69.3)	27 (30.7)	—	1.075 (0.627–1.844)	
**PNC counseling**	**Yes**	153 (72.2)	59 (27.8)	0.962	Ref.	**NS**
	**No**	50 (72.5)	19 (27.5)	—	1.015 (0.553–1.863)	
**EPI counseling**	**Yes**	158 (72.1)	61 (27.9)	0.669	Ref.	**NS**
	**No**	79 (69.9)	34 (30.1)	—	1.115 (0.677–1.836)	
**C. Bivariate Analysis: Unmet Need Variables**						
**Wanted/unwanted pregnancy**	**Yes**	167 (67.6)	80 (32.4)	0.538	Ref.	**NS**
	**No**	71 (71.0)	29 (29.0)	—	0.853 (0.513–1.416)	
**Future plan of child**	**Can't conceive**	2 (12.5)	14 (87.5)	—	Ref.	**S**
	**Undecided/Don't know**	63 (70.0)	27 (30.0)	<0.001*	21.949 (4.847–99.387)	**S**
	**Want child**	99 (68.8)	45 (31.3)	<0.001*	15.400 (3.358–70.618)	**S**
	**No more/No one**	185 (75.8)	59 (24.2)	<0.001*	16.333 (3.471–76.852)	**S**
**D. Multivariate Analysis (Binary Logistic Regression)**						
**Variables**	**Categories**	β-coeff.	p-value	AOR	95% CI	
**Ethnicity**	**Thakuri**	—	—	Ref.	—	
	**Dalit**	0.567	0.331	1.762	(0.562–5.525)	**NS**
	**Janjati**	1.396	0.013	4.037	(1.343–12.139)	**S**
	**Brahmin/Chhetri**	1.023	0.056	2.781	(0.975–7.929)	**NS**
**Husband's occupation**	**Migrant workers**	—	—	Ref.	—	
	**Farmer/Daily wage**	2.127	<0.001	8.389	(3.685–19.101)	**S**
	**Engaged with work**	2.116	<0.001	8.301	(4.534–15.231)	**S**
**Side effects told in facility**	**Yes**	—	—	Ref.	—	
	**No**	0.687	0.007	1.987	(1.211–3.262)	**S**
**FCHV visit last 12 months**	**No**	—	—	Ref.	—	
	**Yes**	0.272	0.423	1.313	(0.675–2.556)	**NS**
**Fieldworker discussed FP**	**No**	—	—	Ref.	—	
	**Yes**	0.274	0.444	1.315	(0.652–2.654)	**NS**

*Significant at p<0.05; b Fisher exact test; S=Significant; NS=Not Significant; COR=Crude Odds Ratio; AOR=Adjusted Odds Ratio; Ref.=Reference category; FP=Family Planning

## References

[1] Ministry of Health and Population [Nepal], New ERA, ICF. Nepal Demographic and Health Survey 2022: Key Indicators Report. Kathmandu: Ministry of Health and Population; 2023. Available from: https://dhsprogram.com/pubs/pdf/PR142/PR142.pdf

[2] Seidu AA Agbaglo E Dadzie LK Ahinkorah BO Ameyaw EK Tetteh JK. Modern contraceptive utilization and associated factors among married and cohabiting women in Papua New Guinea: a population-based cross-sectional study. Contracept Reprod Med. 2020 Nov 18;5(1):22. https://doi.org/10.1186/s40834-020-00125-6 PMid:33292793 PMCid:PMC7672901 10.1186/s40834-020-00125-6 33292793 PMC7672901

[3] Oumer M Manaye A Mengistu Z. Modern contraceptive method utilization and associated factors among women of reproductive age in Gondar City, Northwest Ethiopia. Open Access J Contracept. 2020 Jun 24;11:53-67. https://doi.org/10.2147/OAJC.S252970 PMid:32612400 PMCid:PMC7322113 10.2147/OAJC.S252970 32612400 PMC7322113

[4] Thapa NR. Factors influencing the use of reproductive health services among young women in Nepal: analysis of the 2016 Nepal Demographic and Health Survey. Reprod Health. 2020 Dec;17(1):102. https://doi.org/10.1186/s12978-020-00954-3 PMid:32600458 PMCid:PMC7325020 10.1186/s12978-020-00954-3 32600458 PMC7325020

[5] United Nations Department of Economic and Social Affairs, Population Division. World Family Planning 2022: Meeting the changing needs for family planning: Contraceptive use by age and method. New York: United Nations; 2022. UN DESA/POP/2022/TR/NO. Available from: https://www.un.org/development/desa/pd/sites/www.un.org.development.desa.pd/files/files/documents/2023/Feb/undesa_pd_2022_world-family-planning.pdf

[6] Nepal Demographic and Health Survey Key Indicators Report [Internet]. Available from: https://dhsprogram.com/pubs/pdf/PR142/PR142.pdf

[7] Annual review 2078/79 Birendranagar Municipality [Internet]. Available from: https://birendranagarmun.gov.np/sites/birendranagarmun.gov.np/files/documents

[8] Department of Health Services. Annual Report FY 2076-77. Kathmandu: Ministry of Health and Population, Government of Nepal; 2020.

[9] KC H Shrestha M Pokharel N Niraula SR Pyakurel P Parajuli SB. Women's empowerment for abortion and family planning decision making among marginalized women in Nepal: a mixed method study. Reprod Health. 2021 Feb 4;18(1):28. https://doi.org/10.1186/s12978-021-01087-x PMid:33541377 PMCid:PMC7863411 10.1186/s12978-021-01087-x 33541377 PMC7863411

[10] Chand S Chamroonsawasdi K Vatanasomboon P Chansatitporn N. Assessing the unmet need for modern contraceptives among reproductive-aged women in rural Nepal. J Health Res. 2022;36(3):390-403. https://doi.org/10.1108/JHR-06-2020-0193 10.1108/JHR-06-2020-0193

[11] Wu WJ Maru S. Evaluation of a Community-Based Intervention on Postpartum Contraceptive Utilization in Rural Nepal [12OP]. Obstet Gynecol. 2019 May;133:9. https://doi.org/10.1097/01.AOG.0000559093.08724.c1 10.1097/01.AOG.0000559093.08724.c1

[12] Panta PP Tripathi P Sharma D Amgain K. Knowledge and practice of family planning methods among married women of reproductive age of Chepang community in Benighat VDC of Dhading District. J Karnali Acad Health Sci. 2020;3(1):1-12. https://doi.org/10.3126/jkahs.v3i1.28654 10.3126/jkahs.v3i1.28654

[13] Angdembe MR Sigdel A Paudel M Adhikari N Bajracharya KT How TC. Modern contraceptive use among young women aged 15-24 years in selected municipalities of Western Nepal: results from a cross-sectional survey in 2019. BMJ Open. 2022 Mar 1;12(3):e054369. https://doi.org/10.1136/bmjopen-2021-054369 PMid:35338056 PMCid:PMC8961113 10.1136/bmjopen-2021-054369 35338056 PMC8961113

[14] Joshi AK Tiwari DP Poudyal A Shrestha N Acharya U Dhungana GP. Utilization of family planning methods among postpartum mothers in Kailali District, Nepal. Int J Womens Health. 2020 Jun 24;12:487-94. https://doi.org/10.2147/IJWH.S249044 PMid:32612396 PMCid:PMC7322141 10.2147/IJWH.S249044 32612396 PMC7322141

[15] Pokharel S Khanal G Chapagain S Adhikari K. Use of family planning methods among postpartum mothers in Bharatpur Metropolitan City. MedS Alliance J Med Med Sci. 2022;2:39-43.

[16] Puri MC Huber-Krum S Canning D Guo M Shah IH. Does family planning counseling reduce unmet need for modern contraception among postpartum women: Evidence from a stepped-wedge cluster randomized trial in Nepal. PLoS One. 2021 Mar 26;16(3):e0249106. https://doi.org/10.1371/journal.pone.0249106 PMid:33770114 PMCid:PMC7997001 10.1371/journal.pone.0249106 33770114 PMC7997001

